# Fibronectin aggregates promote features of a classically and alternatively activated phenotype in macrophages

**DOI:** 10.1186/s12974-018-1238-x

**Published:** 2018-08-02

**Authors:** Arend H. Sikkema, Josephine M. J. Stoffels, Peng Wang, Frederike J. Basedow, Robbert Bulsink, Jeffrey J. Bajramovic, Wia Baron

**Affiliations:** 1 University of Groningen, University Medical Center Groningen, Department of Cell Biology, Antonius Deusinglaan 1, 9713 AV Groningen, the Netherlands; 2 Alternatives Unit, Biomedical Primate Research Centre, Lange Kleiweg 161, 2288 GJ Rijswijk, the Netherlands

**Keywords:** Fibronectin, Macrophage, Microglia, Multiple sclerosis

## Abstract

**Background:**

Means to promote endogenous remyelination in multiple sclerosis (MS) benefit from insights into the role of inhibitory molecules that preclude remyelination. Fibronectin assembles into aggregates in MS, which impair oligodendrocyte differentiation and remyelination. Microglia and macrophages are required for complete remyelination and normally switch from a pro-inflammatory classical phenotype upon demyelination to a supportive alternative phenotype during remyelination. Here, we investigated the role of fibronectin aggregates in modulating microglia and macrophage behavior and phenotypes.

**Methods:**

Bone marrow-derived macrophages and microglia from newborn rats were exposed to (a) plasma fibronectin coatings; (b) coatings of deoxycholate-insoluble fibronectin aggregates; (c) interferon-γ (IFNγ) treatment, as an inducer of the pro-inflammatory classically activated phenotype; (d) interleukin-4 (IL-4) treatment, to promote the pro-regenerative anti-inflammatory alternatively activated phenotype; or (e) left unstimulated on uncoated plastic. To examine the in vitro effects of the different stimulations on cell behavior and phenotype, proliferation, phagocytosis, morphology, and pro- and anti-inflammatory features were assessed.

**Results:**

In line with a classically activated phenotype, exposure of microglia and macrophages to both plasma fibronectin and fibronectin aggregates induced an amoeboid morphology and stimulated phagocytosis by macrophages. Furthermore, as observed upon IFNγ treatment, coatings of aggregated, but not plasma fibronectin, promoted nitric oxide release by microglia and macrophages. Remarkably, fibronectin aggregates induced nitric oxide release in an integrin-independent manner. In addition, fibronectin aggregates, but not plasma fibronectin, increased the expression of arginase-1, similarly as observed upon treatment with IL-4. Proteomic analysis revealed that aggregates of fibronectin act as a scaffold for other proteins, including Hsp70 and thrombospondin-1, which may clarify the induction of both pro-inflammatory and anti-inflammatory features in macrophages cultured on fibronectin aggregate, but not plasma fibronectin coatings.

**Conclusions:**

Macrophages and microglia grown on aggregated fibronectin coatings adopt a distinct phenotype compared to plasma fibronectin coatings, showing pro-inflammatory and anti-inflammatory features. Therefore, the pathological fibronectin aggregates in MS lesions may impair remyelination by promoting and/or retaining several classically activated phenotypic features in microglia and macrophages.

**Electronic supplementary material:**

The online version of this article (10.1186/s12974-018-1238-x) contains supplementary material, which is available to authorized users.

## Background

Multiple sclerosis (MS) is a chronic disabling central nervous system (CNS) disease, of which inflammation, demyelination, and neurodegeneration are major pathological features [1]. Myelin regeneration, i.e., remyelination, by oligodendrocyte progenitor cells (OPCs) [2] is apparent in early stages of MS [3]. However, remyelination fails in chronic demyelinated lesions, despite a relative excess of OPCs in the majority of MS lesions [4]. As chronic demyelination leads to neurological disability in MS [5], promoting endogenous remyelination is an attractive therapeutic strategy for structural and functional recovery of MS lesions. However, successful development of such a strategy will require a detailed insight into the mechanism(s) of remyelination failure.

Innate immune activity is an important feature of MS, as signified by activation of resident microglia and invasion of macrophages from the circulation through the disrupted blood-brain barrier [6, 7]. It is estimated that 45% of the macrophages in active MS lesions is derived from microglia [8]. Effector functions of microglia and infiltrated macrophages depend on their phenotype, of which two major utmost categories of a continuum of phenotypes can be discerned. First, the classically activated phenotype is characterized by secretion of reactive oxygen species and pro-inflammatory cytokines, such as tumor necrosis factor-α (TNFα), interleukin-1β (IL-1β), and IL-12. The classically activated phenotype is induced in vitro by stimulation with interferon-γ (IFNγ) or lipopolysaccharide (LPS). Conversely, the alternatively activated regenerative phenotype involves predominantly anti-inflammatory properties, including expression of arginase-1, the mannose receptor, and growth factors such as insulin-like growth factor-1 (IGF-1) and platelet-derived growth factor (PDGF) [9]. The alternative phenotype results from stimulation with interleukin-4 (IL-4) or IL-13. Although the classical/alternative activation concept was originally defined for macrophages, microglia are capable of adopting similar phenotypes [10]. Upon demyelination in the CNS, microglia and macrophages acquire a predominantly classical phenotype, which leads to an increase of phagocytosis as well as expression of pro-inflammatory features, among others, TNFα, IL-1β, and inducible nitric oxide synthase (iNOS, [11–13]). However, at later stages of remyelination, oligodendrocyte differentiation benefits from a switch to the alternative regenerative phenotype, characterized by expression of arginase-1 and the mannose receptor [11–13].

Signals from the micro-environment largely determine the nature of the microglia and macrophage adopted phenotype. Demyelination alters the expression and nature of extracellular matrix (ECM) molecules [14, 15], such as the glycoprotein fibronectin (Fn). Fn is transiently expressed as a dimer in demyelinated lesions, resulting from plasma leakage across the blood-brain barrier, and synthesis by local cells, including astrocytes [15–17]. However, in MS lesions, plasma (pFn) and cell-derived (cFn) assemble into stable aggregates (aFn), which is likely mediated by ongoing inflammation [16]. Aggregated Fn impairs oligodendrocyte differentiation and remyelination [16]. Current evidence suggests that soluble dimeric plasma Fn (pFn), which is transiently expressed in demyelinated lesions [16–18], promotes several pro-inflammatory functions of microglia [19–23]. Given that remyelination likely benefits from a switch between a pro-inflammatory phenotype of microglia and macrophages upon demyelination to a regenerative phenotype at later stages of remyelination [13], we investigated here how aFn, persistently present in MS lesions, affects microglia and macrophage behavior and phenotypes in vitro. Our data revealed that aFn coatings induce a distinct phenotype of bone marrow-derived macrophages (BMDMs) and microglia compared to pFn, supporting several pro-inflammatory classical features such as an amoeboid morphology and nitric oxide (NO) release, and anti-inflammatory features such as increased arginase-1 expression in macrophages. This increased NO-release of aFn coatings appears to be integrin-independent and is likely due to accumulation of other proteins within the aggregates, including heat shock protein 70 (Hsp70) and thrombospondin-1 (TSP1), which use the aggregates as a scaffold. The ability of aFn to induce and likely sustain distinct classical phenotypic features of macrophages and microglia may further contribute to remyelination failure in MS.

## Methods

### MS tissue

Brain tissues were obtained from the Netherlands Brain Bank. Studies were performed on nine control white matter (CWM, healthy subjects), eight chronic (active) MS lesions [c(a)MS], and nine chronic inactive MS lesions (ciMS). Characteristics and selection of the tissues are described previously [24, 25]. For Western blot analysis, mirrored frozen tissues were homogenized and extracted for protein as described [16, 24].

### Cell culture

#### Microglia

Mixed glial cultures were derived from the cerebrum of newborn Wistar rats (P0-P2) and cultured in Dulbecco’s modified Eagle’s medium (DMEM, Life Technologies) containing 10% fetal bovine serum (FBS, Bodinco) and antibiotics (Life Technologies) for 10–12 days as described [26]. Microglia were shaken off mechanically on an orbital shaker (Innova 4000) for 1 h at 150 rpm and cultured in DMEM containing 10% Fn-free FBS, antibiotics, and rat recombinant macrophage colony-stimulating factor (M-CSF; 10 ng/mL, Peprotech) to ensure maturation of the immature neonatal microglia [27]. Fn was eliminated from the serum by means of a Gelatin Sepharose 4B column (GE Healthcare). Shake-off microglia were cultured for 4 days at 37 °C on 10-cm dishes (1.5–2.0 × 10^6^ cells/dish, Corning). Subsequently, mixed glial culture flasks were shaken overnight at 240 rpm. Floating OPCs were purified by differential adhesion, with the cells adhering to the bottom of the dish being in vast majority microglia. These differential adhesion microglia were cultured in microglia medium for 3 days at 37 °C. Both microglia cultures, obtained from shake-off and differential adhesion, were pooled at the start of the experiments. At this stage, microglia cultures were typically 95% pure, with approximately 4% astrocytes and 1% oligodendrocyte lineage cells as assessed by cell-specific immunocytochemistry (see below) for ionized calcium-binding adaptor molecule-1 (Iba1; Abcam; microglia), aldehyde dehydrogenase 1, member L1 (Aldh1L1; Neuromab; astrocytes), and Olig2 (Millipore; oligodendrocytes), respectively.

#### Astrocytes

The remaining astrocyte monolayer of the mixed glial culture flasks was trypsinized and passaged once before experimental use. Regular immunocytochemistry for glial fibrillary acidic protein (GFAP; Millipore) was performed to assure sufficient purity of the astrocyte cultures (> 97%).

#### Macrophages

The hind legs of newborn Wistar rats (P0-P2) were dissected, and the bone marrow cavity of femora and tibiae was flushed with BMDM medium, containing RPMI-1640 medium (Life Technologies), 10% Fn-free FBS, 1% sodium pyruvate (Life Technologies), and antibiotics. To differentiate myeloid progenitor cells towards macrophages, resuspended BMDMs were incubated for 7–8 days in a medium containing M-CSF (10 ng/mL; Peprotech) on 10-cm dishes (2.0 × 10^6^ cells/dish) [28, 29]. Typical BMDM cultures contained > 95% macrophages, as assessed by immunocytochemistry (see below) for isolectin-B4 (IB4; Invitrogen).

#### Stimulation

Microglia or BMDMs were gently scraped in appropriate medium without M-CSF and plated for experiments on 24-well plates (Nunc; 50,000/well in 500 μL of appropriate medium for 24 h), 8-well Permanox chamber-slides (Nunc; 30,000/well in 400 μL of appropriate medium for 24 h, except NO assays: 100,000/well in 300 μL of appropriate medium for 24 h), or 6-well plates (Corning; 1.0 10^6^/well in 2 mL for 6 h). Wells were pre-coated with pFn, aFn (see below), or TSP1 (20 μg/mL; R&D systems), and after 1 h at 37 °C, cells in uncoated wells were stimulated with either rat recombinant IFNγ (5 U/μL; Peprotech), rat recombinant IL-4 (10 μg/mL; Peprotech), rat recombinant Hsp70/Hsp72 (10 ng/mL; Enzo Life Sciences), or left unstimulated. For the integrin blocking experiments, cells were incubated with anti-integrin β1 (CD29; HA2/5; BD Biosciences), anti-integrin β3 (CD61; F11; BD Biosciences) or anti-integrin β5 (P1F6; Millipore) 30 min (adhesion assay) or 24 h (NO release) prior to the analysis.

### Deoxycholate-insoluble fibronectin aggregates

Deoxycholate (DOC)-insoluble aFn was prepared from primary rat astrocytes stimulated with polyinosinic to polycytidylic acid (50 μg/mL, GE Healthcare) for 2 days or brain tissue, as described [16]. Tissue and ECM deposits were suspended in DOC buffer and incubated on ice for 30 min. Pellets were washed three times in phosphate-buffered saline (PBS) followed by resuspension in PBS using a syringe and 25-gauge needle. Protein concentration was determined by Bradford’s protein assay (BioRad) using bovine serum albumin (BSA) as a standard. Presence of aggregates was confirmed by Western blot. To coat wells, either bovine pFn (Sigma-Aldrich) or astrocyte-derived DOC-insoluble aFn were applied for 3 h at 37 °C, using 5 μg on 8-well Permanox chamber slides wells (Nunc) and 96 well plates, and 50 μg on 6-wells plate wells (Corning). For Western blot analysis, pellets were resuspended in non-reducing sample buffer, while supernatants were first concentrated by TCA precipitation.

### Immunocytochemistry

Cells were fixed with 4% paraformaldehyde (PFA) for 20 min. After a 30-min block with 4% BSA in PBS containing 0.1% Triton X-100 (Sigma-Aldrich), cells were incubated for 60 min with Alexa Fluor© 546-conjugated IB4 (Invitrogen; 1:500) or primary antibodies in 4% BSA. Primary antibodies used were against Iba1 (1:500), Aldh1L1 (1:50), Olig2 (1:1000), and GFAP (1:500; Millipore). Cells were washed three times with PBS and incubated for 25 min with appropriate Alexa Fluor©-conjugated secondary antibodies (1:500; Invitrogen). Nuclei were stained with 4′,6-diamidino-2-phenylindole (DAPI; 1 mg/mL; Sigma), and fluorescence mounting medium (Dako) was added to prevent image fading. Images were analyzed using an Olympus Provis AX70 fluorescent microscope or a Leica TCS SP8 Confocal Laser Scanning Microscope.

### BrdU incorporation assay

Cells were allowed to incorporate 5-bromo-2-deoxyuridine (BrdU) (10 μM; Roche) for 24 h. Cells were fixed in PFA for 20 min and additionally fixed in 5% acetic acid in ethanol for 20 min. BrdU was detected using reagents from the BrdU Labelling and Detection Kit I (Roche) according to the manufacturer’s instructions. The numbers of BrdU-positive nuclei were blindly counted relative to the Iba1- or IB4-positive cells (at least 150 cells per condition).

### Phagocytosis assay

Cells were cultured for 24 h, after which fluorescein isothiocyanate (FITC)-labeled latex beads (1 μm; Polyscience) were added at a dilution of 10:1 beads to cells. Phagocytosis of the beads was allowed to proceed for 1 h, after which cells were fixed in 4% PFA. Cells were incubated with DAPI (1 μg/mL) and Alexa Fluor 568-conjugated IB4 in 4% BSA for 2 h. In a blinded manner, the numbers of phagocytosed beads per cell were counted for 100 cells per condition, taking also into account their morphology.

### Nitric oxide assay

Cells were cultured for 24 h, after which the medium was added to a reagent of 0.01% *N*-(1-Naphthyl)-ethylenediamine dihydrochloride (Fluka Analytical) and 0.001% sulfanilamide (Sigma-Aldrich) in 2 M HCl. The absorbance was measured at 550 nm and the NO concentration determined using a standard curve of sodium nitrite (Fluka Analytical).

### Adhesion assay

Cells were pre-incubated with vehicle or anti-integrin antibodies for 30 min at 37 °C and plated at a density of 100,000 cells per well in 96-well plates pre-coated with pFn or aFn. Cells were left to adhere for 1 h at 37 °C. Adherent cells were fixed with ice-cold methanol for 15 min and stained with a 20 mg/mL crystal violet solution (Sigma-Aldrich) for 10 min. After washing, the retained crystal violet was dissolved in 1% SDS followed by measuring the optical density at 570 nm. One hundred thousand cells in a 1.5-mL reaction vial underwent the same steps as the cells that were plated (100% control).

### Arginase assay

Arginase activity was assessed with an arginase assay kit (Abnova) using an adapted protocol based on the manufacturer’s instructions. Briefly, cell pellets were lysed in 100 μL (10 mM Tris-HCl; pH 7.4) supplemented with protease inhibitor without EDTA (Complete; Roche) and 0.1% Triton X-100. After 30 min of gentle agitation at RT, 5 μL MnCl_2_ was added to half of each sample followed by 10 min incubation at 55 °C. MnCl_2_ was subsequently added to the control samples. Arginine substrate buffer was added to the MnCl_2_-primed samples. After incubation for 2 h at 37 °C, substrate buffer was added to the control samples and urea production was measured by reading the optical density at 430 nm.

### TLR4 activation assay

Human endothelial kidney (HEK293) cells were co-transfected with TLR4/MD2/CD14-encoding constructs (InvivoGen) using Polyfect (Qiagen). After selection, TLR-encoding cells were transfected with a reporter vector expressing luciferase under the control of an NF-κB-responsive promoter (pNifty2-luc; InvivoGen). Stably transfected clones were selected and used in bioassays. Cells were plated in pre-coated 96-wells plates at a density of 100,000 cells/well and incubated for 16 h at 37 °C. Cells were lysed in Steady Glo luciferase buffer (Promega), and bioluminescence was measured using a Packard 9600 Topcount Microplate Scintillation & Luminescence Counter (Packard Instrument). As a positive control for NF-κB-mediated activation (i.e., for the presence of the pNifty2-luc vector), 25 ng/ml TNFα (Peprotech) was used (data not shown). LPS (InvivoGen; 1 ng/mL) was used as a positive control for TLR4-mediated activation.

### Real-time quantitative polymerase chain reaction (real-time qPCR)

Cells were cultured for 6 h, after which total RNA was extracted using the RNeasy Micro Kit (Qiagen) followed by reverse transcription (reagents from Thermo Fisher Scientific) according to the manufacturer’s instructions. Real-time qPCR was performed using the Applied Biosystems 7900HT Real-Time PCR System using specific primers (Table 1). Gene expression levels were analyzed using the 2^−ΔΔct^ method, with normalization against hydroxylmethylbilane synthase (HMBS).

**Table 1:** Primer sequences used for real-time, quantitative PCR

	Forward primer	Reverse primer
TNFα	ATGGGCTGTACCTTATCTACTC	GTATGAAATGGCAAATCGGCT
IL-1β	GAAGAATCTATACCTGTCCTGTG	TCTTTGGGTATTGTTTGGGA
IL-12	CTTTGAAGAACTCTAGGTGG	CTTGAGGGAGAAGTAGGAATGG
Arginase-1	ATATCTGCCAAGGACATCGT	ATCACTTTGCCAATTCCCAG
HMBS	CCGAGCCAAGCACCAGGAT	CTCCTTCCAGGTGCCTCAGA
GAPDH	CATCAAGAAGGTGGTGAAGC	ACCACCCTGTTGCTGTAG

### Lactate dehydrogenase and MTT assay

Cells were cultured for 24 h, after which the medium (lactate dehydrogenase (LDH) assay, Roche) and cells (3-(4,5-dimethylthiazol-2-yl)-2,5-diphenyltetrazolium bromide; MTT assay) were analyzed as described [16].

### Proteomics study

DOC-insoluble Fn aggregates were subjected to SDS-PAGE under reducing conditions. Gel lanes were cut in small pieces followed by in gel trypsin digestion. Peptides were extracted and analyzed by liquid chromatography coupled to a high-resolution LTQ Orbitrap XL mass spectrometer (Thermo Fisher Scientific) using ESI as an ion source. Peptide identification was performed with PEAKS software (release 8.5). Of the positive hits, only proteins that are known or predicted to be secreted according the protein atlas were taken into account.

### Western blot analysis

Cells were sonicated or lysed in lysis buffer containing 150 mM sodium chloride, 50 mM Tris-HCl and 5 mM EDTA supplemented with 1% Triton X-100, and protease inhibitor cocktail. Samples were subjected to SDS-PAGE. After transfer of the proteins to PVDF (Immobilon-FL, Millipore) and blocking with 50% Odyssey blocking buffer in PBS the membranes were incubated overnight at 4 °C with primary antibodies against iNOS (1:250; BD Biosciences), arginase-1 (1:500; BD Biosciences) and actin (1:1000; Sigma). Appropriate secondary IRDye-conjugated antibodies were applied for 1 h followed by detection on the Odyssey Infrared Imaging system (Li-Cor Biosciences). Quantification was performed with Scion Image software.

### Statistical analysis

Data were analyzed using SPSS and GraphPad Prism and are reported as mean ± standard error of the mean (SEM) of 2–12 experiments. Results of the morphology, Western blot, NO release, arginase activity, real-time qPCR, LDH, and MTT analyses are presented as a relative to the unstimulated cells (ctrl, set to 100% or 1 in each independent experiment). Statistical analysis was performed with a one sample *t* test when the relative values of the conditions were calculated. When values of two means were compared, statistical significance was calculated by a Student *t* test, and when more than two means were compared, by one-way analysis of variance (ANOVA), followed by Newman-Keuls Multiple Comparison Test post-test. For categorical variables (morphology), logistic regression was performed to compare proportions between the different conditions. *p* values lower than 0.05 were considered statistically significant.

## Results

### Fibronectin aggregates and plasma fibronectin tend to promote proliferation of microglia, but not of bone marrow-derived macrophages

**Fig. 1:**
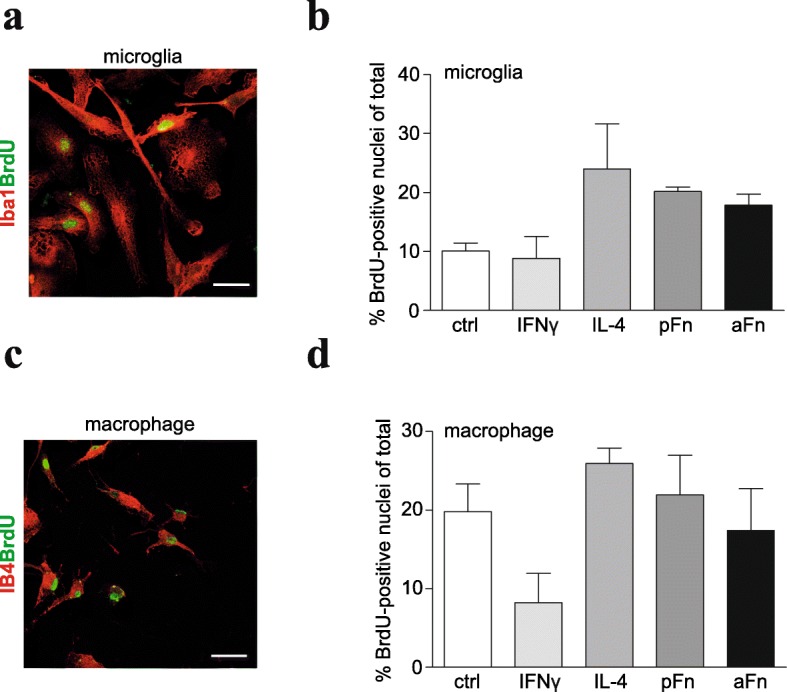
Fibronectin aggregates and plasma fibronectin tend to promote proliferation of microglia. Microglia (**a**, **b**) or bone marrow-derived macrophages (BMDMs, **c**, **d**) were left unstimulated (ctrl), cultured on plasma fibronectin (pFn) or fibronectin aggregates (aFn), or treated with interferon-γ (IFNγ) or interleukin-4 (IL-4). Subsequently, proliferation was determined by allowing the cells to incorporate BrdU for 24 h. BrdU-positive cells (green) that also expressed Iba1 (red, microglia, **a**) or isolectin-B4 (red, IB4, macrophages, **c**) were counted. Compared to control microglia culturing on pFn and aFn coatings tend to enhance proliferation of microglia, similarly as observed upon IL-4 treatment (**b**). Conversely, compared to control macrophages, proliferation of BMDMs on pFn and aFn coatings was hardly affected, while IFNγ tends to decrease BMDM proliferation (**d**). Bars in **b** and **d** represent mean percentages of cells incorporating BrdU from three (microglia) to four (macrophage) independent experiments. Error bars show standard errors of the mean. Statistical analyses were performed using one-way ANOVA (not significant). Scale bar is 10 μm

During CNS demyelination, microglia and infiltrating macrophages increase their numbers by proliferation [30], thereby presumably maximizing their effector functions. In addition, coatings of pFn, which is transiently upregulated upon CNS demyelination [15–17], provide signals that enhance proliferation of microglia via integrin β1 [31]. To assess whether aFn, which is typically present in MS lesions [16], also contributes to such an expansion, we analyzed the effect of aFn and pFn coatings on proliferation of microglia and macrophages, using a BrdU incorporation assay (Fig. 1a, c). To this end, microglia or BMDMs were cultured on either uncoated wells, and wells coated with pFn or aFn. Proliferation of Iba1-positive microglia increased approximately twofold when cultured on both pFn and aFn coatings (Fig. 1b, *p* = 0.09), which is consistent with the previous findings for pFn coatings [31]. Importantly, to eliminate a potential contribution of pFn, normally present in serum, microglia and BMDMs were cultured in Fn-depleted serum. Furthermore, to represent the classical and alternative phenotypes, cells were also stimulated with IFNγ or IL-4, respectively. For microglia, IL-4 tended to increase proliferation (approx. 2.5-fold), in line with previous observations [32], whereas IFNγ hardly, if at all altered proliferation (Fig. 1b). Proliferation of unstimulated BMDMs was more prominent compared to unstimulated microglia (Fig. 1b vs Fig. 1d). When the cells were grown on pFn or aFn coatings, proliferation of the IB4-positive BMDMs was not markedly altered (Fig. 1d), in contrast to the substantial increase seen in case of microglia (Fig. 1b). In addition, IL-4 did not alter the proliferation of BMDMs, whereas IFNγ tended to reduce their proliferation (Fig. 1d, *p* = 0.05). Hence, both pFn and aFn tended to promote proliferation of microglia, but not of BMDMs.

### Fibronectin aggregates and plasma fibronectin increase the number of amoeboid microglia and bone marrow-derived macrophages

**Fig. 2:**
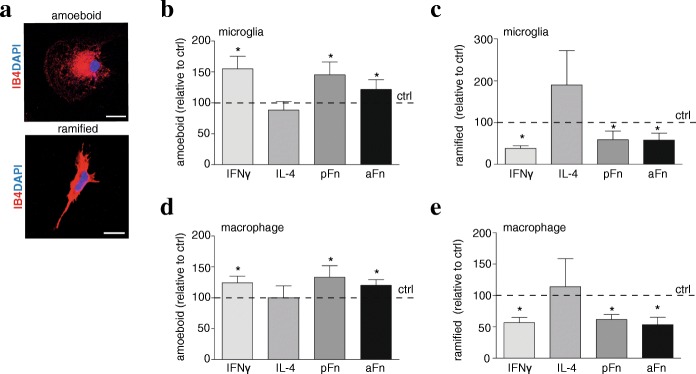
Fibronectin aggregates and plasma fibronectin shift microglia and bone marrow-derived macrophage morphologies towards amoeboid. Microglia (**a**–**c**) or bone marrow-derived macrophages (BMDMs, **d**, **e**) were left unstimulated (ctrl), cultured on plasma fibronectin (pFn) or fibronectin aggregates (aFn), or treated with interferon-γ (IFNγ) or interleukin-4 (IL-4). After 24 h, microglia (**a**–**c**) or BMDMs (**d**, **e**) were immunostained with isolectin-B4 (IB4), and morphologies of 100 cells per condition were scored as “amoeboid,” “ramified,” or “other,” with examples of control microglia shown in **a**. Dashed horizontal lines represent values of control cells set at 100% for each independent experiment. Note that the proportion of microglia and BMDMs with an amoeboid morphology was promoted by IFNγ treatment, pFn or aFn coatings relative to control cells. Bars represent mean values of each condition relative to control cells (set at 100% for each independent experiment, horizontal line) from four independent experiments. Error bars show the standard error of the mean. Statistical analyses were performed using logistic regression for each experiment separately, and representative statistical outcomes are summarized in the graph (*p < 0.05). Scale bar is 10 μm

Classically activated microglia and BMDMs are characterized by a flat and rounded amoeboid morphology, whereas alternatively polarized cells are predominantly ramified with elongated extensions (Fig. 2a) [33]. To investigate whether aFn induces a morphology consistent with either phenotype, we performed immunocytochemistry, using IB4 as a marker, and blindly classified the morphology. The data revealed that both unstimulated control microglia and BMDMs displayed for a major part an amoeboid morphology. These findings are in line with previous observations, which also revealed that culture conditions, and specifically culture serum, slightly activate microglia and BMDMs towards the classical phenotype [34]. Following culturing of the cells on pFn and aFn coatings, a significant increase, relative to unstimulated control cells, was observed in the fraction of both microglia and BMDMs displaying an amoeboid morphology, an effect compatible to IFNγ treatment (Fig. 2b, d). Simultaneously, the proportion of ramified microglia and BMDMs decreased on pFn or aFn substrates, as well as following IFNγ treatment (Fig. 2c, e). In contrast, and rather unexpectedly [35], exposure to IL-4 did not significantly change the morphology of microglia and BMDMs, compared to that seen for unstimulated cells (Fig. 2b–e). Notably, the relative fractions of microglia and BMDMs classified as being of “other” morphology, i.e., cells that could not be classified as either “ramified” or “amoeboid,” did not change in any of the examined conditions. Accordingly, pFn and aFn coatings induce an amoeboid morphology of microglia and BMDMs, in line with the classically activated phenotype.

### Fibronectin aggregates and plasma fibronectin selectively affect phagocytosis in microglia versus bone marrow-derived macrophages

**Fig. 3:**
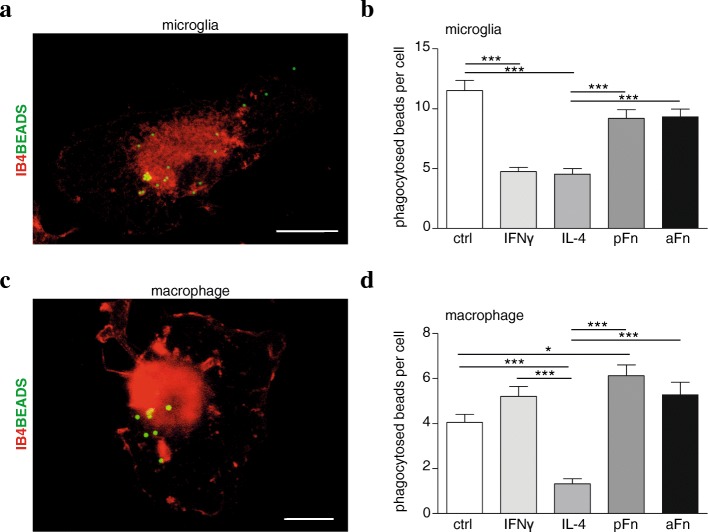
Fibronectin aggregates and plasma fibronectin promote phagocytosis by bone marrow-derived macrophages, but not by microglia. Microglia (**a**, **b**) or bone marrow-derived macrophages (BMDMs, **c**, **d**) were left unstimulated (ctrl), cultured on plasma fibronectin (pFn) or fibronectin aggregates (aFn), or treated with interferon-γ (IFNγ) or interleukin-4 (IL-4). Then, microglia and BMDMs were allowed to phagocytose fluorescently labeled latex beads for 1 h. The numbers of ingested beads (green) were counted in isolectin-B4-positive cells (IB4, red). Bars represent mean numbers of phagocytosed beads. Error bars show the standard error of the mean. Representative graphs of duplicate experiments are shown. Statistical analyses were performed using one-way ANOVA, followed by a Newman-Keuls Multiple Comparison test (*p < 0.05; ***p < 0.001). Scale bar is 10 μm

Phagocytosis is a functional property of microglia and macrophages and can be triggered by integrin-mediated signaling [36], with the integrin being a receptor for Fn. To examine the effect of Fn coatings on phagocytosis, the ingestion of fluorescently labeled latex beads was determined (Fig. 3a, c). Our results revealed that phagocytosis of latex beads in unstimulated control microglia amounted to 12 ± 1 beads/hour (Fig. 3b). Culturing microglia on pFn or aFn coatings did not markedly alter their phagocytic activity (Fig. 3b). However, exposure of the microglia to IFNγ or IL-4 reduced the phagocytic activity of the cells by approximately 50%, showing a residual ingestion activity of 5 ± 1 beads/hour (Fig. 3b). BMDMs similarly displayed phagocytic activity, but these cells were less active than the microglia, i.e., unstimulated BMDMs ingested 4 beads/hour (Fig. 3d). However, in contrast to microglia, BMDM phagocytic activity tended to increase when the cells were cultured on pFn and aFn, or exposed to IFNγ treatment (Fig. 3d). Similarly, to observations for microglia, phagocytosis by BMDMs was significantly reduced upon exposure to IL-4 (Fig. 3d vs Fig. 3b).

Receptor-mediated phagocytosis of myelin by macrophages and microglia is stimulated by IFNγ and is considered a feature of classically activated amoeboid cells [37]. Indeed, upon morphologically classifying each phagocytosing cell, a predominant amoeboid morphology was apparent for either cell type, i.e., 80 ± 6% and 64 ± 3% for microglia and BMDMs, respectively. However, the numbers of beads phagocytosed per cell by amoeboid microglia did not significantly differ from the numbers of beads phagocytosed per cell by microglia of ramified or intermediate morphologies. In contrast, ramified BMDMs phagocytosed on average significantly less beads than BMDMs of amoeboid or intermediate morphologies (*p* < 0.01). At the different conditions, however, the numbers of beads phagocytosed by ramified BMDMs were similar to the numbers indicated above (Fig. 3d), when morphology was not taken into account. Therefore, differences in numbers of beads phagocytosed by microglia or BMDMs do not merely signify dynamic changes that are reflected by their morphology. These results indicate that, in contrast to treatment with IFNγ and IL-4, culturing microglia on aFn or pFn substrates does not markedly reduce phagocytosis. In addition, our data indicate that phagocytosis by BMDMs is promoted when the cells are grown on pFn and aFn coatings, similarly as observed upon IFNγ treatment.

### Fibronectin aggregates, but not plasma fibronectin, promote release of nitric oxide by microglia and bone marrow-derived macrophages

**Fig. 4:**
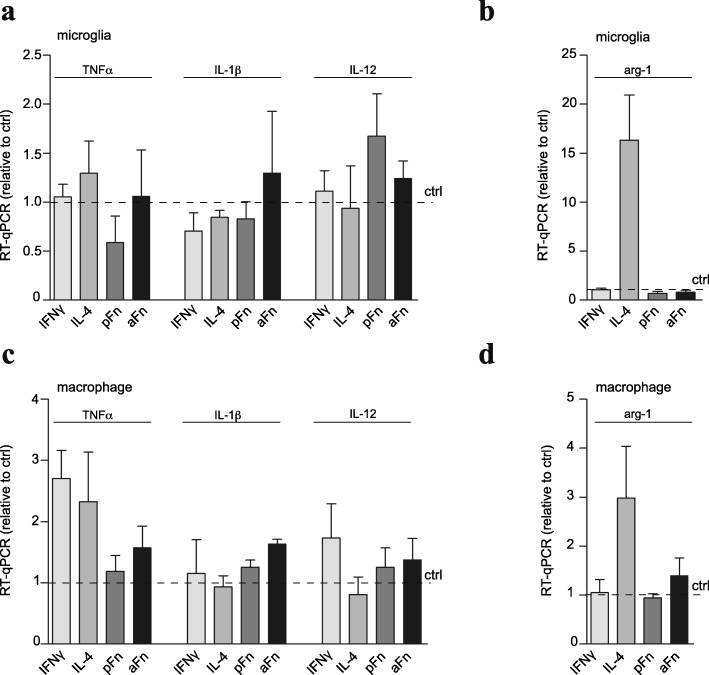
Fibronectin aggregates and plasma fibronectin do not significantly alter cytokine and chemokine gene expression, representative of classically or alternatively activated phenotypes, in microglia and bone marrow-derived macrophages. Microglia (**a**, **b**) or bone marrow-derived macrophages (BMDMs, **c**, **d**) were left unstimulated (ctrl), cultured on plasma fibronectin (pFn) or fibronectin aggregates (aFn), or treated with interferon-γ (IFNγ) or interleukin-4 (IL-4) for 6 h, after which total RNA was extracted. Cytokine and chemokine gene expression levels were analyzed using quantitative real-time PCR against pro-inflammatory markers for the classically activated phenotype (**a**, **c**, tumor necrosis factor-α (TNFα), interleukin-1β (IL-1β) and interleukin-12 (IL-12)) and an anti-inflammatory marker for the alternatively activated phenotype (**b**, **d**, arginase-1 (arg-1)) against HMBS (shown) and GAPDH (not shown, but yielding comparable findings). Bars represent mean expression levels versus control (set at 1 for each independent experiment, horizontal line) from three independent experiments. Error bars show the standard error of the mean. Statistical analyses were performed using the one-sample *t* test when compared to control (*p < 0.05; **p < 0.01)

**Fig. 5:**
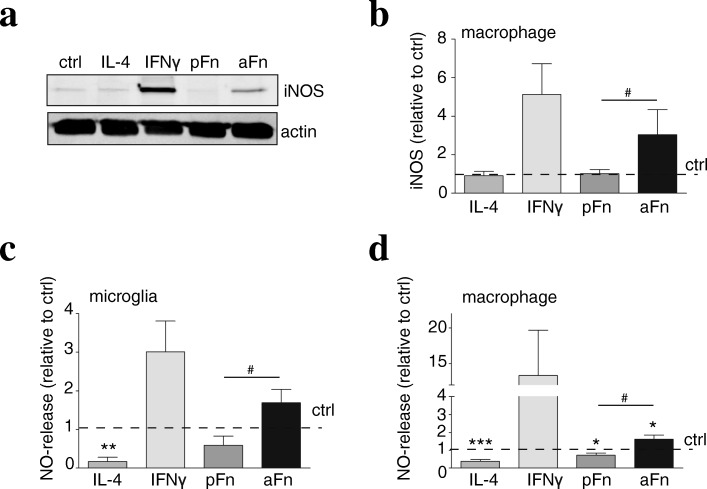
Fibronectin aggregates, but not plasma fibronectin, induce iNOS expression by bone marrow-derived macrophages*.* Microglia (**c**) and bone marrow-derived macrophages (BMDMs, **a**, **b**, **d**) were left unstimulated (ctrl), cultured on plasma fibronectin (pFn) or fibronectin aggregates (aFn), or treated with interferon-γ (IFNγ) or interleukin-4 (IL-4). Then, the expression of iNOS (**a**, **b**) and nitric oxide (NO) levels (**c**, **d**), markers for classically activated microglia and BMDMs, were analyzed as described in materials and methods. Note that aFn, but not pFn, increased both iNOS (**a**, **b**, *n* = 4–5), along with an increased NO release (**c**, **d**, *n* = 5–12). Bars represent mean values of each condition relative to control cells (set at 1 for each independent experiment, horizontal line). Error bars show the standard error of the mean. Statistical analyses were performed using the one-sample *t* test when compared to control (*p < 0.05; **p < 0.01; ***p < 0.001). A student *t* test was performed to compare pFn with aFn (^#^*p* < 0.05)

A typical feature of the classical phenotype is the expression of pro-inflammatory cytokines, whereas alternative polarization generally induces the expression of anti-inflammatory factors. Therefore, we next examined gene expression of TNFα, IL-1β, and IL-12 as representatives of the classically activated microglia/macrophage phenotype. To mark anti-inflammatory alternative polarization, we analyzed mRNA levels of arginase-1 [9]. Culturing microglia on either pFn or aFn did not change the expression of pro-inflammatory cytokines compared to unstimulated control microglia (Fig. 4a). Surprisingly, pro-inflammatory cytokine gene expression was neither induced upon IFNγ exposure (Fig. 4a), in spite of an amoeboid morphology that was promoted at the same conditions (Fig. 2b). Of note, when the microglia (and BMDMs) were exposed to lipopolysaccharide (LPS), a more potent inducer of the pro-inflammatory phenotype, in combination with IFNγ, TNFα mRNA levels were markedly increased compared to control (31.2 ± 12.2 and 29.6 ± 12.2 fold change, respectively). Anti-inflammatory gene expression was not enhanced by microglia cultured on pFn and aFn (Fig. 4b). Furthermore, IL-4 treatment markedly, but not significantly, enhanced anti-inflammatory arginase-1 gene expression (Fig. 4b). Similarly as observed for microglia, culturing BMDMs on pFn and aFn neither induced a clear expression of a pro-inflammatory or anti-inflammatory gene signature (Fig. 4c, d). Rather, exposure to IFNγ slightly upregulated the pro-inflammatory cytokine genes TNFα and IL-12 (Fig. 4c), whereas IL-4 tended to enhance the expression of the arginase-1 gene (Fig. 4d). Because synthesis of reactive oxygen species, such as NO is another important pro-inflammatory and MS-relevant characteristic of microglia and macrophages [38], we next analyzed iNOS expression on pFn and aFn coatings. Western blot analyses revealed that whereas unstimulated control BMDMs hardly express iNOS, culturing cells on aFn tended to enhance iNOS expression levels (Fig. 5a, b). Remarkably, culturing BMDMs on pFn did not enhance iNOS expression compared to unstimulated BMDMs, indicating that aFn and pFn elicit different signals to the cells. In fact, the iNOS expression levels in BMDMs significantly differ between pFn and aFn coatings. As expected, IFNγ, but not IL-4 treatment, reproducibly induced iNOS expression by BMDMs (Fig. 5b). Similar experiments with microglia showed that the expression levels of iNOS for IFNγ-treated microglia were increased compared to control microglia, while iNOS was hardly, if at all, detectable in cells cultured on aFn and pFn coatings (data not shown). Similarly, IFNγ treatment reproducibly increased NO release by microglia into the medium (*p* = 0.07), an enhancement that was more prominent than when the cells were cultured on aFn coatings (Fig. 5c). For BMDMs, NO levels in the culture medium displayed a very similar trend in response to the various treatments/conditions as those observed for microglia. Thus, a reproducible increase in NO release was triggered in cells grown on aFn, but not pFn coatings, while a prominent increase in NO release was measured upon exposure to IFNγ (Fig. 5d). Notably, for both microglia and BMDMs, the release of NO was significantly higher on aFn than on pFn coatings (Fig. 5c, d). To exclude the possibility that NO release by microglia and BMDMs on aFn coatings is a consequence of a cytotoxic effect induced by aFn, we performed LDH and MTT assays at different concentrations of aFn for both cell types. As shown in Additional file 1, aFn did not markedly alter LDH release by microglia and BMDMs compared to unstimulated cells (Additional file 1: Figure S1A, C). Also, microglia viability, as measured by MTT reduction, was comparable to the viability of unstimulated cells (Additional file 1: Figure S1B). BMDM viability was slightly affected on aFn (Additional file 1: Figure S1D), but given a similar LDH release, this may reflect differences in metabolic rate, rather than cytotoxicity. Hence, aFn coatings do not induce significant cytotoxicity of microglia and BMDMs, consistent with previous findings for OPCs [16]. Therefore, these results indicate that aFn coatings, but not pFn coatings, promote a release of NO by microglia and BMDMs.

### Fibronectin aggregates, but not plasma fibronectin, promote arginase expression and activity by bone marrow-derived macrophages

**Fig. 6:**
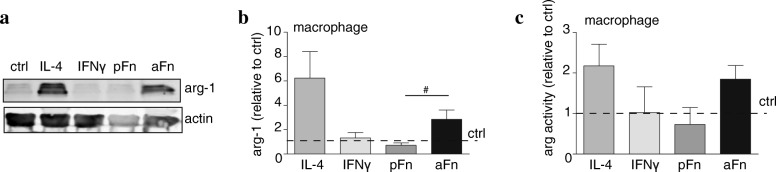
Fibronectin aggregates, but not plasma fibronectin, induce arginase-1 expression by bone marrow-derived macrophages*.* Bone marrow-derived macrophages (BMDMs, **a**–**c**) were left unstimulated (ctrl), cultured on plasma fibronectin (pFn) or fibronectin aggregates (aFn), or treated with interferon-γ (IFNγ) or interleukin-4 (IL-4). Then, the expression of arginase-1 (**a**, **b**) and activity (**c**), indicative for alternative polarization, were analyzed as described in materials and methods. Note that aFn, but not pFn, increased arginase-1 expression (**a**, **b**, *n* = 4–5), along with an increased arginase activity (**c**, *n* = 3). Bars represent mean values of each condition relative to control cells (set at 1 for each independent experiment, horizontal line). Error bars show the standard error of the mean. Statistical analyses were performed using the one-sample t-test when compared to control (not significant). A student t test was performed to compare pFn with aFn (# *p* < 0.05)

As qPCR findings showed a slight tendency of an increased expression of arginase-1 on aFn coatings, we next assessed the arginase-1 protein expression. Notably, iNOS and arginase-1 share the same substrate (i.e., l-arginine) and are opposing markers of classically and alternatively activated microglia/macrophage phenotype respectively [39]. Western blot analyses revealed that culturing cells on aFn coatings (*p* = 0.07) and IL-4 treatment (*p* = 0.09) tended to promote the expression of arginase-1, whereas unstimulated control BMDMs and BMDMs cultured on pFn coatings hardly expressed arginase-1 (Fig. 6a, b). Also, arginase-1 expression in BMDMs was significantly increased on aFn coatings compared to pFn coatings (Fig. 6a, b). These findings indicate that, similar to the induction of iNOS and NO-release, aFn and pFn elicited different signals in BMDMs. Whereas exposure to IL-4 also enhanced arginase-1 expression in microglia, arginase-1 was not detectable upon exposure to aFn and pFn coatings (data not shown). In fact, upon loading equal protein amounts, the arginase-1 levels in microglia were considerably lower than the levels in macrophages, while the levels obtained in cells cultured on aFn and pFn may be beyond the detection limit. To corroborate that the observed enhanced arginase-1 levels in BMDMs on an aFn coating (Fig. 6a, b) was also reflected by an increase in the enzyme’s activity, an arginase activity assay was performed. As shown in Fig. 6c, similar to IL-4 treatment, aFn tended to increase arginase activity by BMDMs compared to unstimulated BMDMs and BMDMs cultured on a pFn coating. For microglia, and consistent with the low expression levels, arginase activity was hardly detectable, even following IL-4 treatment. Thus, in the presence of aFn coatings, but not pFn coatings, BMDMs display features of both classically and alternatively activated phenotypes. Intriguingly, the effect of aFn differed from pFn coatings, indicating that different receptors may be involved.

### Fibronectin aggregates induce NO release in an integrin β1, β3, and β5-independent manner

**Fig. 7:**
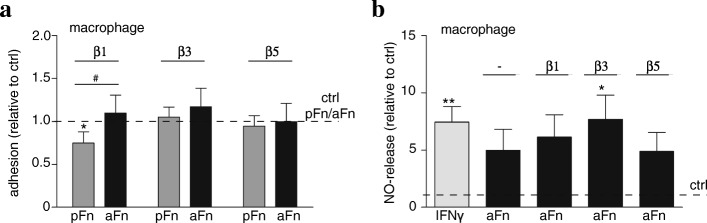
Fibronectin aggregates induced release of nitric oxide by bone marrow-derived macrophages is integrin β1, β3, and β5 independent. **a** Bone marrow-derived macrophages (BMDMs) were pre-incubated with vehicle or the indicated functional blocking anti-integrin antibodies, plated on plasma fibronectin (pFn) or fibronectin aggregates (aFn) and subjected to an adhesion assay. Note that pre-incubation with anti-integrin β1 antibodies reduced the adhesion of BMDMs to pFn, but not to aFn. **b** BMDMs were left unstimulated (ctrl), cultured on aFn, or treated with interferon-γ (IFNγ). Cells cultured on aFn were left untreated or treated with functional anti-integrin β1, β3, or β5 antibodies. Then, nitric oxide (NO) levels in the culture medium were analyzed as described in materials and methods. Note that the aFn-mediated increase in NO release is integrin-independent. Bars represent mean values of each condition relative to control cells (set at 1 for each independent experiment, horizontal line) from four (adhesion) to five (NO assay) independent experiments. Error bars show the standard error of the mean. Statistical analyses were performed using the one-sample *t* test when compared to control (*p < 0.05; **p < 0.01). A student *t* test was performed to compare pFn with aFn (^#^*p* < 0.05)

pFn and aFn differ in that aFn contains both pFn and cFn. cFn, but not pFn, may contain an EIIIA domain (EDA in human), which has been shown to bind to Toll-like receptor 4 (TLR4) on inflammatory cells [40]. We therefore next analyzed whether aFn coatings were able to induce TLR4-mediated responses. While HEK293 cells, transfected with TLR4/MD2/CD14, showed a robust response to the TLR4 agonist LPS, TLR4 was not activated on pFn and aFn coatings (Additional file 2: Figure S2). However, LPS retained, although a bit decreased, the ability to activate TLR4 on HEK293 cells grown on Fn coatings, indicating that pFn and aFn coatings slightly interfered with TLR4 activation (Additional file 2: Figure S2). As integrins are major cell surface receptors for Fn [41], we next analyzed whether adhesion of BMDMs to pFn and aFn is integrin-dependent. As shown in Fig. 7a, a functional blocking antibody against integrin β1 inhibited BMDM adhesion to pFn, but not aFn. Functional blocking antibodies against integrins β3 and β5 hardly, if at all affect adhesion of BMDMs to either substrate (Fig. 7a), indicating that these integrins are not essential for BMDM adhesion to pFn and aFn coatings. Moreover, the aFn-induced NO release could not be overcome by functional blocking antibodies (Fig. 7b), indicating that macrophage integrins β1, β3, and β5 are dispensable. In addition to binding sites for integrins, Fn also contains binding sites for other proteins, such as fibrin and collagen [41], to which microglia/macrophages may bind. In addition, aFn may act as scaffold for other proteins, and as such may harbor many proteins, which we set out to determine next.

### MS lesions-derived fibronectin aggregates act as a scaffold for Hsp70 and thrombospondin

**Fig. 8:**
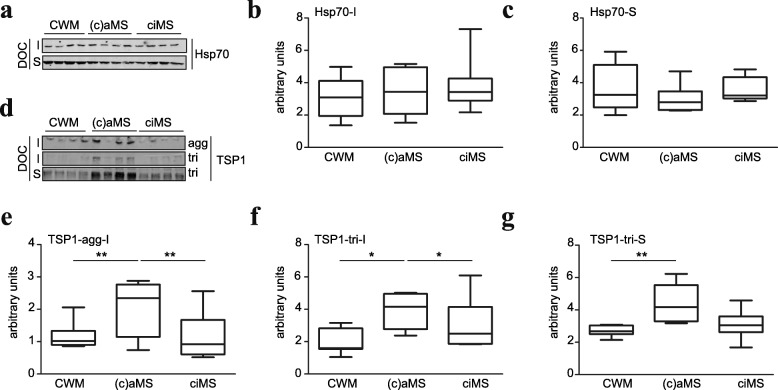
Hsp70 and thrombospondin-1 are present in MS lesion-derived fibronectin aggregates. Homogenates of human control white matter (*n* = 9), (chronic) active MS lesions ((c)sMS, *n* = 8) and chronic inactive MS lesions (ciMS, *n* = 9) were subjected to deoxycholate (DOC) (in)solubility assays (**a**–**g**). Hsp70 (**a**–**c**) and thrombospondin-1 (TSP1, **d**–**g**) levels in DOC-insoluble (I, containing Fn aggregates) and DOC-soluble (S, containing Fn dimers) fractions were analyzed by Western blot under non-reducing conditions. Note that TSP1 is enriched in (c)aMS lesions compared to CWM and ciMS lesions (**d**–**g**), while Hsp70 is equally present (**a**–**c**). In addition, TSP1 is tightly associated with the aggregates (agg, **d**, **e**). Box plots represent arbitrary intensity values of each condition. Statistical analyses were performed using an one-way ANOVA, followed by a Newman-Keuls Multiple Comparison test (*p < 0.05, **p < 0.01)

To reveal other players associated with aFn that may modulate the phenotype of BMDMs, we performed proteomic analysis on the DOC-insoluble fibronectin aggregates obtained from deposits of activated astrocytes. Proteomic analysis revealed that next to Fn, 17 other proteins were present in the DOC-insoluble rat astrocyte-derived deposit extracts, including ECM protein vitronectin, matricellular proteins TSP1, tenascin-C, CYR61 and connective tissue growth factor, and heat shock proteins Hsp70, Hsp47, and Hsp90 (Table 2). Notably, as aFn is formed extracellularly (our unpublished observations) and to reduce the number of false positive hits, i.e., upon lysis of the astrocytes intracellular proteins may associate with the aggregates, only proteins that are known to be secreted were considered. Western blot analysis revealed that Hsp70 and TSP1 were also present in MS lesion-derived fibronectin aggregates. As shown in Fig. 8a–c, Hsp70 was prominently present in DOC-insoluble fractions that contain aFn, but also in DOC-soluble fractions that do not contain aFn [16], indicating that not all Hsp70 was associated with aFn in MS lesions. Also, similar levels of Hsp70 were measured in control white matter (CWM), chronic active MS lesions [c(a)MS], and chronic inactive MS lesions (ciMS). In contrast to Hsp70, a fraction of TSP1 appeared to be tightly associated with aFn in chronic (active) MS lesions, as it was detectable in the DOC-insoluble fraction at the top of the gel (Fig. 8d, e), i.e., the fraction where aFn is recovered [16]. Furthermore, a minor portion of TSP1 was present as trimer in the DOC-insoluble fraction, likely reflecting TSP1 that was weakly associated with aFn in chronic (active) MS lesions (Fig. 8d, f). In addition, a significant fraction of TSP1 was not associated with aFn in chronic (active) MS lesions, given its abundant presence in the aFn-free DOC-soluble fraction (Fig. 8d, g). In CWM and ciMS lesion homogenates, the levels of TSP1 were less abundant than in (c)aMS lesion homogenates. Hence, TSP1 and Hsp70 may use aFn as a scaffold, and by doing so add to the observed classically and/or alternatively activated features of BMDMs, when cultured on aFn coatings. Indeed, extracellular Hsp70 is suggested to stimulate exposure of cell surface receptors that are also present on microglia/macrophages, including TLR2, TLR4, and CD40 [42, 43]. Similarly, TSP1 ligands, such as CD36 and CD47, are also functionally expressed on macrophages [44].

**Table 2:** Predicted secreted proteins present in rat astrocyte-derived fibronectin aggregates

Protein	Score	#spec	#pep	#uniq	%spec	%cov
Serine protease HTRA1	170.1	21	6	6	10.5	11.9
Protein CYR61	168.6	8	8	8	16.7	17.2
Cell migration-inducing hyaluronan-binding protein	159.3	15	6	6	3.9	4.9
Heat shock protein HSP 90-beta (HSP90β)	154.6	9	5	2	4.7	7.2
Connective tissue growth factor	136.1	5	5	5	11.6	13.0
Thrombospondin 1	123.9	15	4	4	3.4	3.7
Ttrifunctional enzyme subunit beta, mitochondrial	120.2	5	4	4	8.0	7.4
Vitronectin	117.9	14	4	3	7.8	5.9
Serpin H1 (HSP47)	116.9	3	2	2	3.7	7.0
Tenascin C	116.3	5	3	3	1.7	1.4
Fibronectin	112.7	4	3	2	1.6	1.4
Serine (Or cysteine) peptidase inhibitor, clade C, member 1	112.2	13	2	2	3.7	4.7
78 kDa glucose-regulated protein (HSP70 protein 5, HSPA5)	101.4	4	4	3	4.6	8.3
Coagulation factor V	97.9	2	2	2	1.2	1.2
Elastin microfibril interfacer 1	95.1	7	3	3	4.1	3.3
Complement component 4A	91.1	5	2	2	1.1	1.0
Complement C4B	91.1	5	2	2	1.1	1.0
Complement C4	91.1	5	2	2	1.1	1.0

*Spec* specific, *pep* peptide, *unq* unique, *cov* coverage, *#* number

### Hsp70 increases iNOS expression, while both Hsp70 and thrombospondin induce arginase expression by bone marrow-derived macrophages

**Fig. 9:**
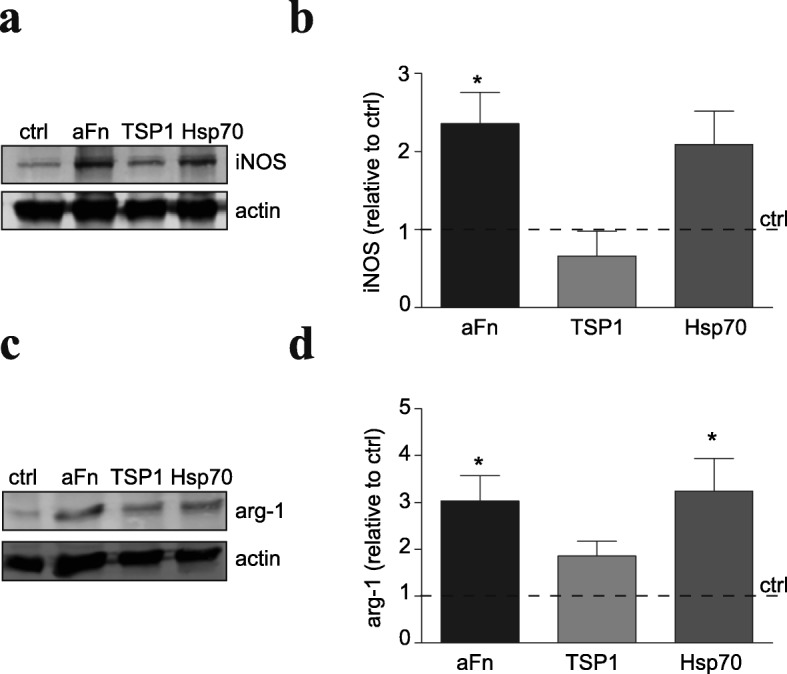
Hsp70 increases iNOS expression, while both Hsp70 and thrombospondin-1 induce arginase-1 expression by bone marrow-derived macrophages. Bone marrow-derived macrophages (BMDMs, **a**–**d**) were left unstimulated (ctrl), cultured on fibronectin aggregates (aFn) or thrombospondin-1 (TSP1) or treated with Hsp70. Then, the expression of iNOS (**a**, **b**) and arginase-1 (**c**, **d**) was analyzed by Western blotting. Note that both aFn and Hsp70 increase iNOS and arginase-1 expression, while TSP1 induced only arginase-1 expression. Bars represent mean values of each condition relative to control cells (set at 1 for each independent experiment, horizontal line) from four independent experiments. Error bars show the standard error of the mean. Statistical analyses were performed using the one-sample *t* test when compared to control (*p < 0.05)

To assess whether Hsp70 and/or TSP1 modulate macrophage phenotype, BMDMs were plated on a TSP1 coating or were exposed to Hsp70. Western blot analysis demonstrated that, to an extent similar as in case of aFn, extracellular Hsp70 significantly increased the expression of iNOS in BMDMs, relative to control unstimulated BMDMs (Fig. 9a, b). Remarkably, exposure to extracellular Hsp70 also enhanced the expression of the alternatively activated marker arginase-1 by threefold in BMDMs, i.e., similar as aFn coatings (Fig. 9c, d). Culturing BMDMs on a coating of TSP1 induced an almost twofold increase in the arginase-1 expression (Fig. 9d, *p* = 0.07), while iNOS expression remained similar to control (Fig. 9b). Hence, the observed aFn coating-induced dual classically and alternatively activated features in BMDMs, which differ from those observed on pFn coatings, are likely mediated via aFn-associated proteins, rather than Fn itself.

## Discussion

In this study, we characterized phenotypes of microglia and BMDMs cultured on coatings of structural variants of Fn, an ECM protein that aggregates in MS lesions, thereby inhibiting remyelination [16]. Our data revealed that several microglia- and macrophage-related pro-inflammatory features were induced to a similar extent by both pFn and aFn, including a predominantly amoeboid morphology of microglia and BMDMs, increased proliferation of microglia, and enhanced phagocytosis by BMDMs. Therefore, aggregation of Fn, which results from strong, non-covalent protein-protein interactions and is defined by DOC-insolubility [45], may particularly represent a continuous state of pFn signaling, which activates several properties of microglia and macrophages. However, the aFn matrix also expressed an additional effect compared to pFn, in that it caused a stimulation of NO release by microglia and BMDMs, and an enhanced arginase-1 expression and activity by BMDMs. Accordingly, our data indicate that aFn promotes a distinct phenotype of macrophages, and likely also of microglia, displaying features of both the classically and alternatively activated phenotype.

Initially, we hypothesized that this additional effect of aFn may be related to the potential presence of *cellular* Fn in aFn, which contains the alternatively spliced EIIIA and EIIIB domains that are absent from plasma-derived pFn [41]. The EIIIA domain is a ligand for the α9β1 receptor, and activation of this receptor promotes NO production in a human colon adenocarcinoma cell line [46]. Also, Fn fragments that contain the EIIIA domain stimulate TLR4 [40], known to promote pro-inflammatory polarization of microglia and macrophages [47]. Hence, these previous findings suggest a potential underlying mechanism for the aFn-mediated increase in NO levels. However, aFn coatings hardly, if at all activated TLR4, and the effect of aFn coatings on NO release was integrin β1-independent. Rather, as revealed by proteomic analysis, the data indicate that the distinct effect of aFn compared to pFn coatings is mediated by proteins, such as Hsp70 and TSP1, that may exploit aFn as a scaffold. In fact, our findings demonstrate that the expression of TSP1 is increased in chronic (active) MS lesions and tightly associated with aFn. TSP1 harbors an Fn binding site [44], and its receptors, CD36 and CD47, are functionally expressed on microglia and macrophages and may have opposing effect on macrophage polarization [48–51]. Our findings showed that TSP1 coatings promoted arginase-1, but not iNOS expression by macrophages, which is consistent with its suggested role in limiting pro-inflammatory effects [48]. On the other hand, the function of TSP1 is highly dependent on its expression levels and on which domain is functional in a given biological setting. At low levels, TSP1 may only interact with CD47 and promote alternative activation in macrophages by inhibiting the production of pro-inflammatory features, including the production of NO, IL-12, and IL-1, while at high levels, TSP1 may also bind to CD36, resulting in the release of IL-1 and IL-6 [48–50], but also of the anti-inflammatory marker IL10 [51]. Extracellular Hsp70 is suggested to act as an endogenous agonist of TLR2, TLR4, and CD40 [42, 43] and, with its confirmed presence in the DOC-insoluble MS lesion-derived aFn fraction, may add to the mixed phenotype features, promoted by aFn coatings, while its action via TLR4 is excluded. Indeed, similar to aFn, Hsp70 treatment enhanced both iNOS and arginase-1 expression by BMDMs. Whether other identified aFn-associated proteins, i.e., tenascin C and connective tissue growth factor, both known to bind to Fn [52, 53], affect microglia and BMDM activation remains to be determined. Hence, by acting as scaffold for several proteins that are ligands for receptors present on microglia and macrophages, pro-inflammatory and/or anti-inflammatory features may be induced by aFn.

Next to using aFn as a scaffold and their interference with glial cell behavior, the identified proteins in the DOC-insoluble Fn aggregates may play a role in aggregation per se. For example, vitronectin inhibits Fn matrix assembly via its heparin-binding domain [54]. Also of interest in this respect is the aFn-association of Hsp70, Hsp47, and Hsp90β, which are found extracellular and are linked to ECM remodeling. Thus, Hsp70 increases collagen and Fn expression via transforming growth factor-β1 (TGF-β1) signaling [55], Hsp47 act as a receptor for collagen fibrillogenesis [56], and extracellular Hsp90β binds directly to Fn and increases the formation a DOC-insoluble Fn matrix [57]. Of interest, Hsp70 and Hsp90β are associated with MS pathology [58, 59]. A role of these Hsps in Fn aggregation in MS lesions remains to be determined.

Previous studies showed that soluble pFn may also promote NO synthesis [21], as well as synthesis of pro-inflammatory cytokines, such as TNFα [21, 22]. Our seemingly opposing findings, which revealed that pFn coatings, in contrast to soluble pFn [21], do not promote NO release and that both pFn and aFn coatings do not induce mRNA expression of pro-inflammatory cytokines, can likely be attributed to the different signaling properties of Fn coatings, as opposed to those of soluble Fn. Immobilized Fn coatings, which probably better than soluble Fn mimic the deposited Fn matrix in MS lesions, enforce clustering of integrin receptors, and may also bind to different receptors, i.e., both integrins and others [60]. Indeed, the upregulation of, among others, TNFα by soluble pFn is mediated via TLR4, whereas the present results demonstrated that coatings of pFn and aFn do not activate TLR4. This may also explain why pFn did not markedly enhance phagocytosis by microglia in our studies, which is considered to be mediated by TLR4 [22]. Furthermore, we investigated relatively naïve microglia and BMDMs, whereas priming of microglia and BMDMs may enhance their responsiveness to Fn. For instance, LPS-primed microglia respond to soluble pFn by additionally increasing IL-1β production [23].

Microglia and BMDMs are distinct cell types [61], and recent evidence indicates that their effector functions in MS may be different [62–64]. To clarify whether both cell types respond similarly to aFn, we directly compared activation profiles from microglia and BMDMs, obtained in parallel from the same pool of neonatal rats. Although microglia and BMDMs respond similarly with respect to morphology, NO release, and cytokine gene expression, several differences were also apparent. First, microglia expressed a more prominent potential of phagocytosis than BMDMs, in line with previous reports [10, 37]. This likely sustains the notion that phagocytosis by microglia occurs without priming in CNS physiology [65, 66]. In addition, BMDMs enhanced pro-inflammatory cytokine gene expression more readily upon IFNγ treatment than microglia, whereas microglia responded to IL-4 with a much greater increase of arginase mRNA expression, the latter not being reflected at the protein level. Thus, together with a more pronounced NO release by BMDMs upon IFNγ treatment, and arginase-1 expression and activity upon exposure to IL-4 and on aFn coatings, these findings support the concept that macrophages are immune mediators per se, whereas microglia display more plasticity, with diverse, non-immunological roles in the healthy brain [61–63].

The overall effects of microglia and macrophage activation by aFn on remyelination remain to be determined. Phagocytosis of myelin debris by macrophages is thought to promote remyelination [67, 68], but phagocytosis of neuronal debris can also enhance myelin and axonal destruction by the adaptive immune system [69]. Similarly, NO has favorable effects on immune activation in MS, but also mediates oligodendrocyte and myelin injury [38]. In general, however, the early phase of remyelination benefits from a classically activated microglia and macrophage phenotype, whereas oligodendrocyte differentiation and myelin production are promoted by a switch to the alternatively activated phenotype during later stages [13]. Hence, transient Fn expression upon demyelination [15–17] likely contributes to the temporal classical phenotype of microglia and macrophages, whereas in MS lesions Fn persists as aggregates, and as a consequence, microglia and macrophages may retain and/or gain some pro-inflammatory features that may interfere with the regenerative alternatively activated phenotype that is required for remyelination [13]. Indeed, in active MS lesions, a major subset of macrophages display classically and alternatively activated markers, indicating an intermediate activation status [70]. Hence, in addition to directly inhibiting OPC differentiation, unfavorable microglia and macrophage polarization may represent an indirect mechanism underlying aFn-mediated impairment of remyelination.

## Conclusion

This study revealed that aFn promotes features in microglia and macrophages that are distinct from pFn, likely via co-association of distinct proteins within the aggregates. Given that alternatively activated microglia and macrophages are required for successful remyelination [13], the persistent expression of aFn in MS lesions may impair remyelination by promoting and/or retaining features of the classically activated phenotype in microglia and macrophages.

## Additional files

Additional file 1: **Figure S1.** Fibronectin aggregates do not induce cell cytotoxicity as determined by lactate dehydrogenase release and an MTT assay. Microglia (**a**, **b**) or bone marrow-derived macrophages (BMDMs, **c**, **d**) were left unstimulated (ctrl) or cultured on fibronectin aggregates (aFn; 2, 5, or 10 μg, respectively) for 24 h, followed by measurements of lactate dehydrogenase (LDH) release in the culture medium (**a**, **c**) and of MTT reduction (**b**, **d**). Bars represent means versus control (set at 1 for each independent experiment, horizontal line). Error bars show the standard error of the mean. Statistical analyses were performed using the one-sample *t* test when compared to control (**p < 0.01). (TIF 11900 kb)

Additional file 2: **Figure S2.** Fibronectin aggregates and plasma fibronectin do not activate TLR4 on HEK293 cells. HEK293 cells transfected with TLR4/MD2/CD14 were plated on uncoated plastic (ctrl) or on coated 96 wells plates that were coated with plasma (pFn) or aggregated fibronectin (aFn). Cells were left untreated or treated with the TLR4 agonist LPS. Bars represent mean fold increase in bioluminescence. Error bars show the standard error of the mean. Statistical analyses were performed using a one-way ANOVA, followed by a Newman-Keuls Multiple Comparison test (*p < 0.05, **p < 0.01, ***p < 0.001). (TIF 7670 kb)

## Abbreviations

aFn: Fibronectin aggregates
Aldh1L1: Aldehyde dehydrogenase 1, member L1
ANOVA: Analysis of variance
BMDM: Bone marrow-derived macrophage
BrdU: 5-Bromo-2-deoxyuridine
BSA: Bovine serum albumin
c(a)MS: Chronic (active) multiple sclerosis lesion
cFn: Cell-derived fibronectin
ciMS: Chronic inactive MS lesion
CNS: Central nervous system
DAPI: 4′,6-Diamidino-2-phenylindole
DMEM: Dulbecco’s modified Eagle’s medium
DOC: Deoxycholate
ECM: Extracellular matrix
FBS: Fetal bovine serum
FITC: Fluorescein isothiocyanate
Fn: Fibronectin
GFAP: Glial fibrillary acidic protein
HMBS: Hydroxylmethylbilane synthase
Hsp70: Heat shock protein 70
IB4: Isolectin-B4
Iba1: Ionized calcium-binding adaptor molecule-1
IFNγ: Interferon-γ
IGF-1: Insulin-like growth factor-1
IL-1β: Interleukin-1β
IL-4: Interleukin-4
iNOS: Inducible nitric oxide synthase
LDH: Lactate dehydrogenase
LPS: Lipopolysaccharide
M-CSF: Macrophage colony-stimulating factor
MS: Multiple sclerosis
MTT: [3-(4,5-Dimethylthiazol-2-yl)-2,5-diphenyltetrazolium bromide
NO: Nitric oxide
OPC: Oligodendrocyte progenitor cell
PBS: Phosphate-buffered saline
PDGF: Platelet-derived growth factor
PFA: Paraformaldehyde
pFn: Plasma fibronectin
SEM: Standard error of the mean
TGF-β1: Transforming growth factor-β1
TLR4: Toll-like receptor 4
TNFα: Tumor necrosis factor-α
TSP1: Thrombospondin-1
