# Supplementary figures and images for: Fibronectin aggregates promote features of a classically and alternatively activated phenotype in macrophages
Source: J Neuroinflammation. 2018 Aug 2;15:218. doi: 10.1186/s12974-018-1238-x (PMC6091019; doi:10.1186/s12974-018-1238-x)

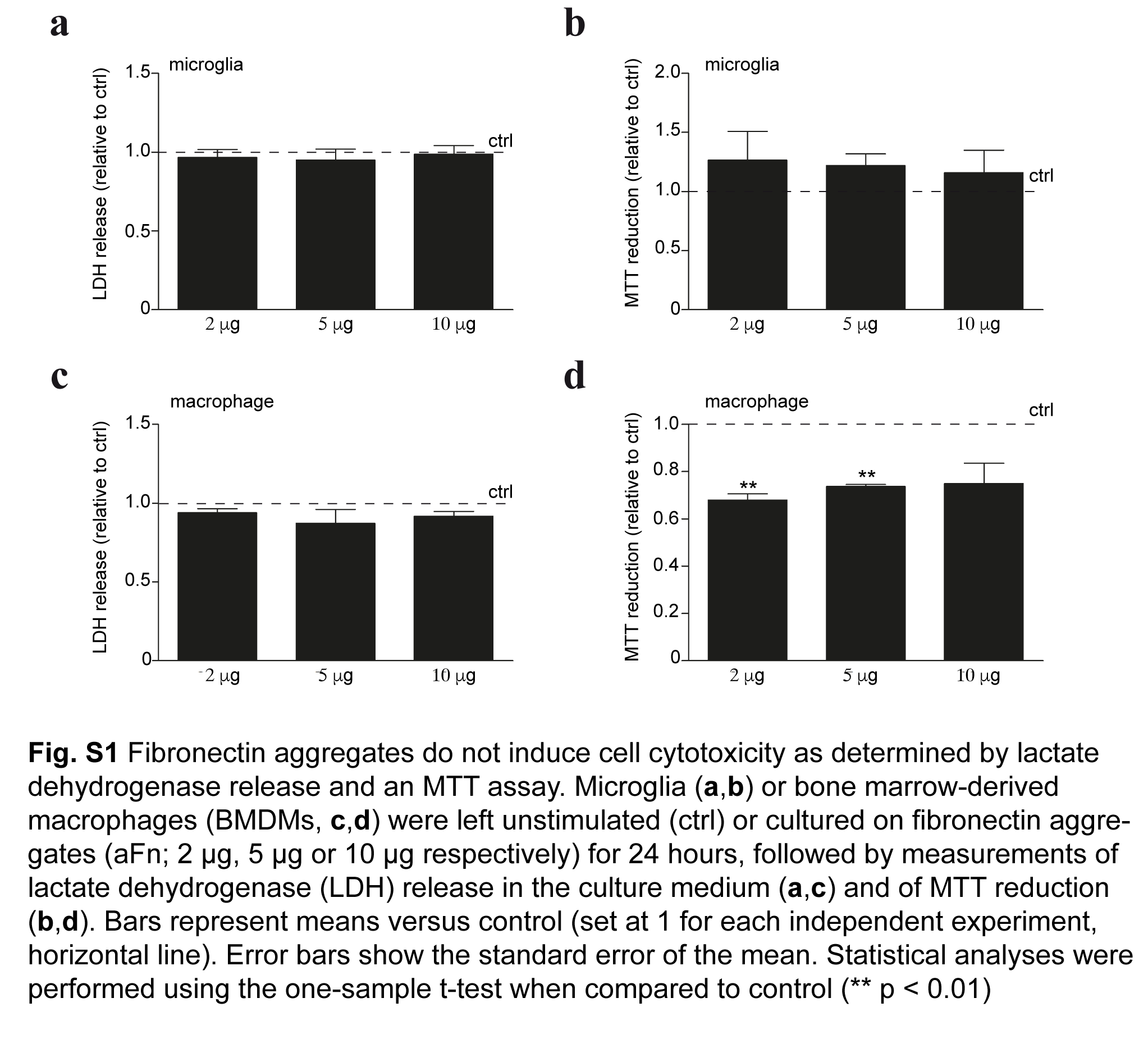

Supplement: Supplementary file 1 — Figure S1. Fibronectin aggregates do not induce cell cytotoxicity as determined by lactate dehydrogenase release and an MTT assay. Microglia (a, b) or bone marrow-derived macrophages (BMDMs, c, d) were left unstimulated (ctrl) or cultured on fibronectin aggregates (aFn; 2, 5, or 10 μg, respectively) for 24 h, followed by measurements of lactate dehydrogenase (LDH) release in the culture medium (a, c) and of MTT reduction (b, d). Bars represent means versus control (set at 1 for each independent experiment, horizontal line). Error bars show the standard error of the mean. Statistical analyses were performed using the one-sample t test when compared to control (**p < 0.01). (TIF 11900 kb) [file 12974_2018_1238_MOESM1_ESM.tif]

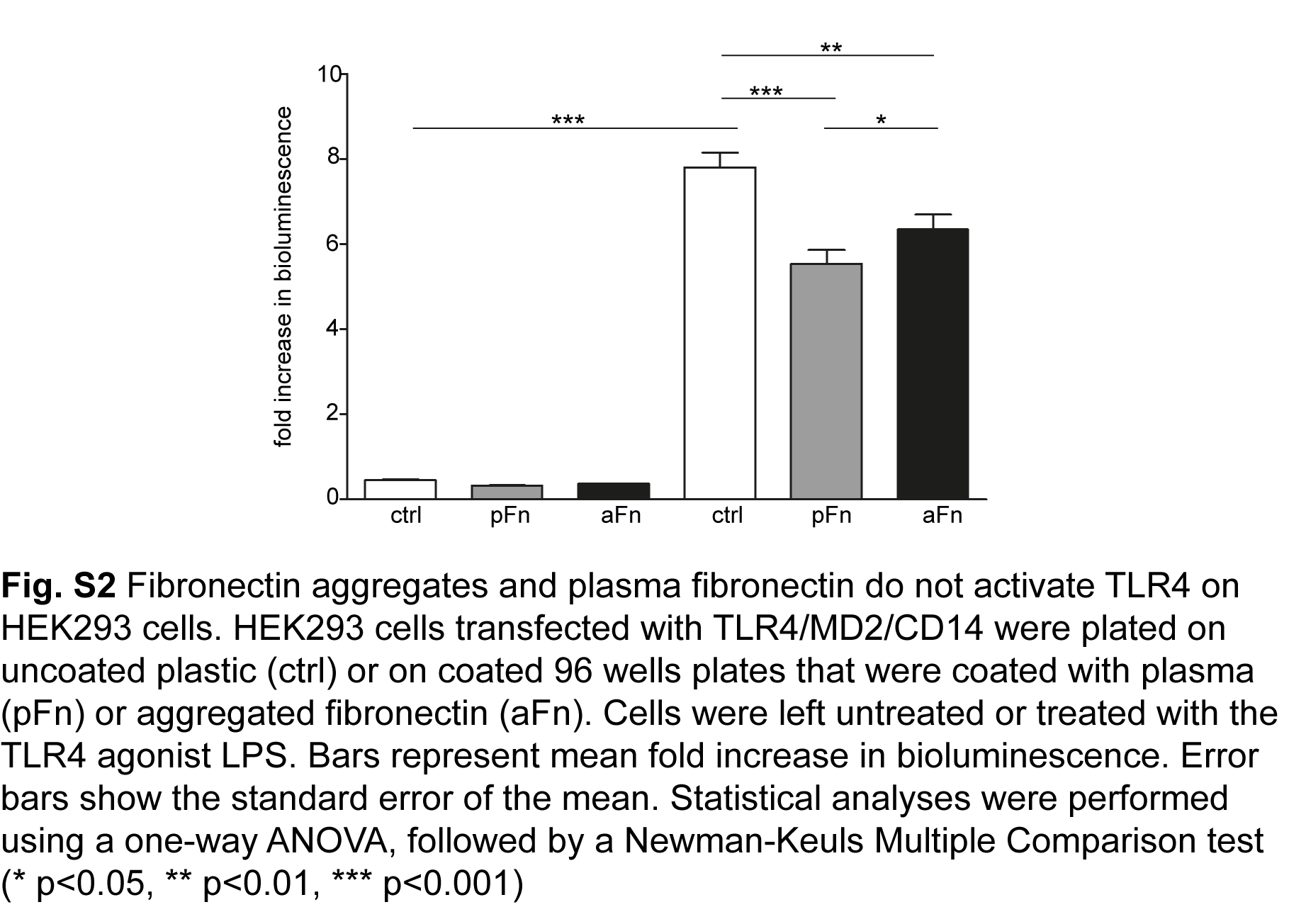

Supplement: Supplementary file 2 — Figure S2. Fibronectin aggregates and plasma fibronectin do not activate TLR4 on HEK293 cells. HEK293 cells transfected with TLR4/MD2/CD14 were plated on uncoated plastic (ctrl) or on coated 96 wells plates that were coated with plasma (pFn) or aggregated fibronectin (aFn). Cells were left untreated or treated with the TLR4 agonist LPS. Bars represent mean fold increase in bioluminescence. Error bars show the standard error of the mean. Statistical analyses were performed using a one-way ANOVA, followed by a Newman-Keuls Multiple Comparison test (*p < 0.05, **p < 0.01, ***p < 0.001). (TIF 7670 kb) [file 12974_2018_1238_MOESM2_ESM.tif]
